# Congo Red Interactions with Curli-Producing *E*. *coli* and Native Curli Amyloid Fibers

**DOI:** 10.1371/journal.pone.0140388

**Published:** 2015-10-20

**Authors:** Courtney Reichhardt, Amy N. Jacobson, Marie C. Maher, Jeremy Uang, Oscar A. McCrate, Michael Eckart, Lynette Cegelski

**Affiliations:** 1 Department of Chemistry, Stanford University, Stanford, California 94305, United States of America; 2 Protein and Nucleic Acid Facility, Stanford University, Stanford, California 94305, United States of America; INRA, FRANCE

## Abstract

Microorganisms produce functional amyloids that can be examined and manipulated *in vivo* and *in vitro*. *Escherichia coli* assemble extracellular adhesive amyloid fibers termed curli that mediate adhesion and promote biofilm formation. We have characterized the dye binding properties of the hallmark amyloid dye, Congo red, with curliated *E*. *coli* and with isolated curli fibers. Congo red binds to curliated whole cells, does not inhibit growth, and can be used to comparatively quantify whole-cell curliation. Using Surface Plasmon Resonance, we measured the binding and dissociation kinetics of Congo red to curli. Furthermore, we determined that the binding of Congo red to curli is pH-dependent and that histidine residues in the CsgA protein do not influence Congo red binding. Our results on *E*. *coli* strain MC4100, the most commonly employed strain for studies of *E*. *coli* amyloid biogenesis, provide a starting point from which to compare the influence of Congo red binding in other *E*. *coli* strains and amyloid-producing organisms.

## Introduction

*E*. *coli* and *Salmonella* species assemble extracellular adhesive amyloid fibers termed curli that mediate cell-surface and cell-cell interactions and serve as an adhesive and structural scaffold to promote biofilm assembly and other community behaviors [1–4]. Curli are among a growing list of functional microbial amyloids that emphasize Nature’s ability to coordinate the assembly of amyloid fibers to promote community behavior and function. Amyloid fibers contribute to sporulation in *Streptomyces* [5] and to adhesion and biofilm formation in *E*. *coli* [1,4] as well as in *Salmonella* species [2], *B*. *subtilis* [6], *S*. *aureus* [7], *and S*. *mutans* [8], and others [8,9]. Curli and other amyloid fibers have important roles in modulating the viscoelastic properties of biofilms. This property has been identified in rheological studies of natively produced curli-containing pellicle (biofilm formed at the air-liquid interface) [10] and in studies of *in vitro* formed biofilm-like materials [11]. In *E*. *coli*, curli biogenesis requires specific molecular machinery encoded by the *csgBA* and *csgDEFG* operons [3]. *In vivo* polymerization of the major curli subunit CsgA into β-sheet-rich amyloid fibers requires the nucleator protein, CsgB [3]. CsgG is an outer membrane protein [12] and CsgE and CsgF are assembly factors required for the stabilization and transport of CsgA and CsgB to the cell surface [13,14]. Thus, in contrast to the undesired and alternative protein folding events that lead to amyloid formation in human amyloid diseases including Alzheimer’s, Parkinson’s, and Huntington’s diseases [15], bacteria harness dedicated machinery in order to direct the assembly of amyloid fibers at their cell surface for function.

As amyloid, curli share some general structural, biochemical, and biophysical properties with other functional amyloids and disease-related amyloids. Structurally, amyloid fibers are comprised of polypeptides rich in β-sheet secondary structure in which individual β-strands are primarily aligned perpendicular to the fiber axis [16,17]. Amyloid fibers share biochemical properties: they are resistant to SDS [18] and proteases [19] and they bind the classic amyloid dyes, Congo red (CR) and thioflavin T [20]. CR was the dye first used to identify amyloid in tissue specimens and remains a benchmark to identify the presence of amyloid through its detectable fluorescence upon binding to amyloid or its birefringence under polarized light. CR binding to β-amyloid has been studied extensively and reviewed recently, and it is used to ultimately confirm diagnoses of Alzheimer’s diseases through post-mortem staining of brain tissue [21].

Curli production among *E*. *coli* and *Salmonella* strains is often scored qualitatively by the staining of colonies grown in the presence of CR. However, because CR can bind to other cellular features in some bacterial strains including cellulose, care must be taken to consider dye binding as a reliable indicator of amyloid production only among bacterial strains that exhibit curli-dependent CR binding, *i*.*e*. among strains in which curli mutations abolish CR binding [22]. In this way, CR-binding phenotypes have been valuable in studies of curli biogenesis in MC4100, the most well studied *E*. *coli* strain for dissecting curli biogenesis [3]. MC4100 produces curli localized at the cell surface. When grown on CR-containing nutrient agar medium, curliated whole cells bind CR and deplete the dye from the underlying agar. Deletion of the curli chaperone-like protein, CsgF, on the other hand, results in aberrant assembly and fiber mislocalization [12]. In the *csgF* mutant, CR binding is observed in the underlying agar after cells are removed from the agar, which is attributed to the mislocalization of curli fibers away from the cell surface [12]. This phenotype, as well as other phenotypes ascribed to fibers formed in modified genetic backgrounds, has improved our model of curli assembly and the multi-protein curli machinery. The coordinated assembly process emphasizes the importance of examining the structural and biochemical properties of curli when formed natively by *E*. *coli*, when possible, rather than using curli-like fibers polymerized *in vitro* from purified preparations of the major fiber subunit protein, CsgA. Thus, we examined the interactions of curliated whole cells and native curli with the amyloid dye CR.

## Results and Discussion

**Fig 1:**
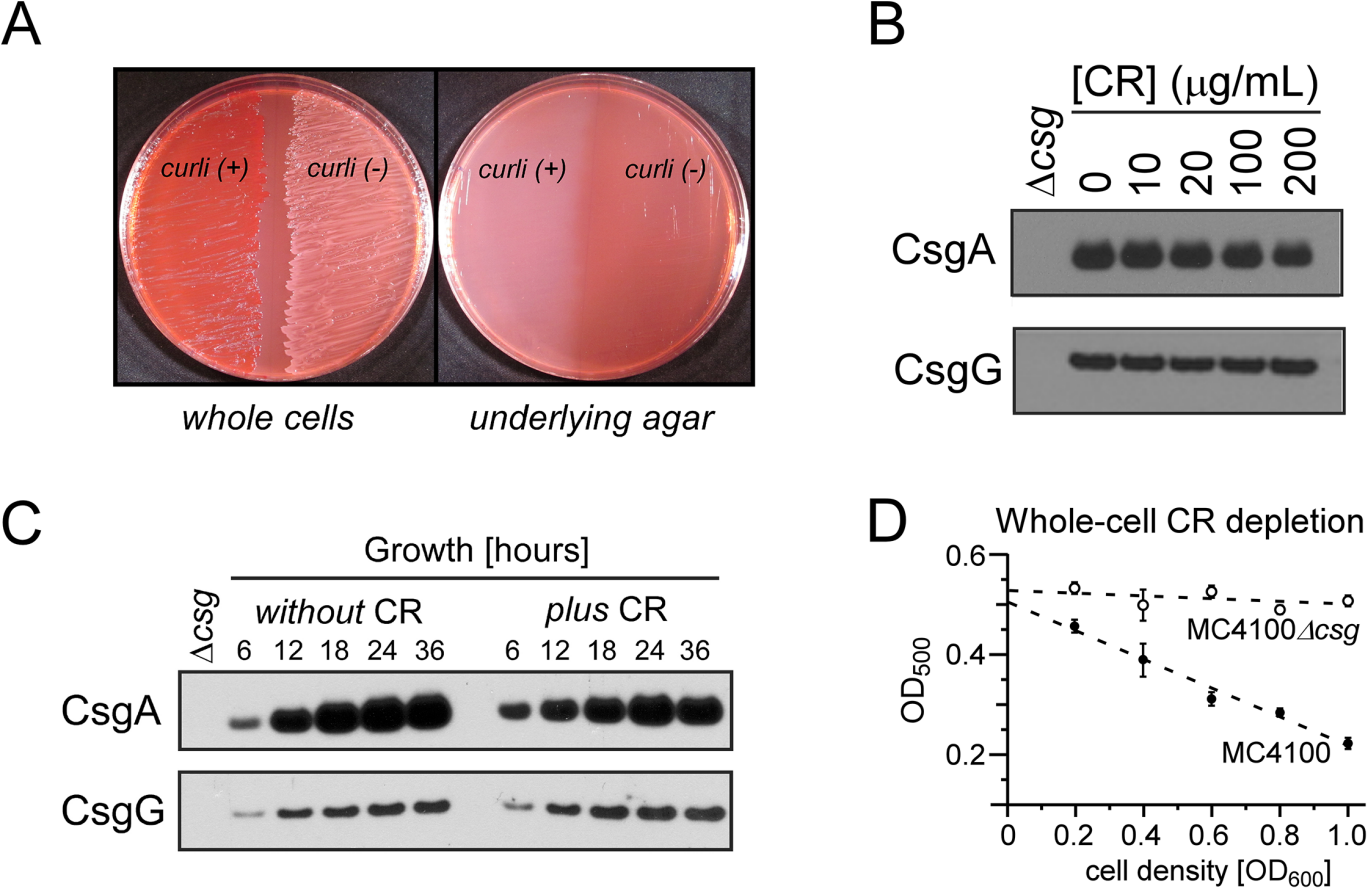
Congo red and *E*. *coli*. (A) Curli-producing *E*. *coli* bind CR when growing on CR-supplemented nutrient agar (left). The depletion of CR from the underlying agar can be observed when cells are removed from the growth plate (right). (B) Supplementation of the agar with CR at concentrations up to 200 μg/mL does not affect curli production on a per cell basis as assessed by Western blot analysis. CsgA is the main curli fiber subunit and CsgG oligomerizes in the outer membrane to form a pore for the transport of curli subunits to the cell surface. (C) Supplementation of the agar with CR also does not influence the typical kinetics of curli production, where more curli are produced as cells enter the stationary phase. (D) A whole-cell CR depletion assay provides a way to evaluate and compare curli production among MC4100 samples. Bacteria were incubated with 10 μg/mL CR and the extent of free CR in the supernatant after centrifugation of CR-bound whole cells was monitored by UV-Vis spectrophotometry. CR depletion from the supernatant is enhanced in a linear fashion as the number of cells and amount of curli are increased. Error bars correspond to the standard deviation of measurements for three separate samples.

CR has been used extensively to supplement nutrient agar as a selection medium to distinguish curli-producing bacteria from non-curliated bacteria when CR-binding is confirmed to be curli-dependent (Fig 1A). Final CR concentrations in indicator plates are typically 10–30 μg/mL. We determined that bacterial growth in CR-containing agar medium with final CR concentrations as high as 200 μg/mL did not alter curli production, as assessed by Western blot analysis with the detection of CsgA and CsgG protein levels of whole-cell samples normalized by cell number (Fig 1B). This assay was also performed for a panel of uropathogenic *E*. *coli*, and we similarly found that CR did not inhibit curli production for these clinically relevant *E*. *coli* strains (S1 Fig) [23,24]. S1 Fig also demonstrates that curli produced by UTI89 and the other clinical isolates grown in the presence of CR required formic acid treatment for depolymerization to run into the gel for subsequent detection, a standard requirement for amyloid fibers. Western blot analysis as a function of growth time also revealed that CR does not influence the kinetics of curli production *in vivo* (Fig 1C). The appreciated time-dependence of curli production on agar can be noted in the curli protein Western blot profiling, where curli production is upregulated as cell density increases from 6 through 36 hours. Thus, although CR has been shown to inhibit the formation of amyloid fibers by some amyloidogenic proteins *in vitro* [25], we demonstrate that CR does not inhibit the biogenesis of curli *in vivo* during growth on YESCA agar.

Curliated whole cells grown normally on YESCA agar without CR have been noted for their ability to bind CR upon incubation with a CR solution [26,27]. We characterized this phenomenon quantitatively using MC4100 whole cells. Increases in CR depletion tracked linearly with bacterial concentration (Fig 1D). As the density of MC4100 was increased, more CR was depleted from the solution. Curli mutant cells, MC4100Δ*csg*, did not exhibit significant CR binding and depletion. Thus, relative cell-associated curli levels in MC4100 can be determined by a CR depletion assay. This provides a convenient and rapid way to assay and compare curli production across whole-cell samples that may be advantageous as a higher throughput assay without requiring immunoblotting or fiber purification protocols.

Previous studies with isolated native curli [3] and other amyloid fibers, notably β-amyloid [28], have demonstrated the absorbance and fluorescence spectral changes that occur when CR binds to curli and amyloids. UV-Vis spectra exhibit an increase in CR intensity and a red shift when CR is bound to curli[3]. In addition, CR exhibits very little detectable fluorescence in solution, but fluoresces when bound to curli [3,22]. We extended the biophysical analyses of native curli to examine CR binding using Surface Plasmon Resonance (SPR).

The use of SPR to examine the binding of CR to curli required the identification of a suitable regeneration condition in order to remove CR from immobilized fibers after compound association and dissociation events during the SPR measurement (S2 Fig). Of all the conditions tested to remove CR bound to native curli fibers, a few successful regeneration conditions were identified: 100% DMSO; 50% DMSO/10 mM NaOH; and 100% DMF. Conditions that did not remove CR from curli fibers included: acetone, dichloromethane, ethyl acetate, 2-propanol, tetrahydrofuran, and toluene. Additionally, a solution of 100 mM HCl that was employed for the regeneration of curli-like fibers fibrillized *in vitro* [29] was not sufficient to remove CR from our native curli fibers. We selected 50%DMSO/10mM NaOH as the regeneration condition for our SPR experiments as it was most compatible with the SPR instrument and protocols.

**Fig 2:**
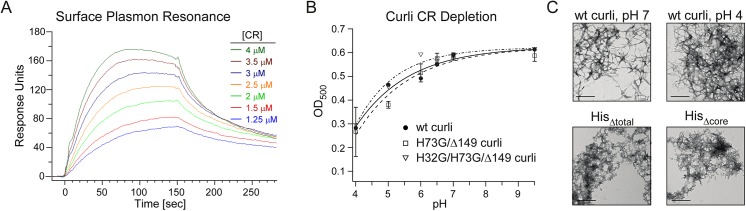
Interactions of CR with isolated curli. (A) Surface plasmon resonance experiments performed with immobilized curli revealed that CR binding is relatively weak; CR binds to curli with an approximate k_d_ of 2.8 μM. (B) A CR depletion assay allowed facile examination of CR binding to isolated curli, similar to the whole-cell assay performed in Fig 1. The data demonstrate that CR binding to curli is pH dependent and is not dependent on histidine in the CsgA sequence. (C) Curli produced by wild-type MC4100 are comparable to those formed by the histidine mutants and are not influenced by changes in pH, as demonstrated by electron microscopy.

The association and dissociation kinetics of CR as a function of concentration are presented in Fig 2A. CR association gradually increased during the association phase from 0 to 150 s as CR was introduced at the specified concentration through continuous flow over immobilized curli fibers on the SPR chip for the duration of the association phase (Fig 2A). During the dissociation phase, CR was not present in the buffer and a gradual decline in response units (RU) during the next 150 s revealed the dissociation of CR from the immobilized fibers, yielding a final residual response of about 50 RU. Thus, a fraction of CR remained bound to curli and was only removed after the regeneration step. The complete removal of CR and regeneration of curli was achieved with a solution of 50%DMSO/10mM NaOH. Binding to curli was unaffected by repeated regeneration and identical response curves were obtained whether the experiments were performed as a function of increasing or decreasing CR concentration. The equilibrium binding constant was obtained using the Langmuir model (A+L ←→ AL) by non-linear curve fitting of the Langmuir binding isotherm, R_eq_ = (k_a_ [A]/ (k_a_ [A] + K_d_)) * R_max_, where R_eq_ is the shift in SPR angle at equilibrium (or “Response units”, RU) and R_max_ is the maximum analyte binding capacity of the surface in RU. The equilibrium constant, K_d_, for CR binding to curli was determined to be 2.8 μM, as calculated from the association and dissociation rates. This value is similar to those reported for CR binding to other amyloids including Aβ11–28 (1–10 μM) [30].

CR binding to β-amyloid fibers formed *in vitro* has been reported to be pH-dependent [30]. To determine if CR binding to curli fibers displayed a similar pH-dependence, we adapted the whole-cell CR depletion assay to monitor the depletion of CR resulting from CR binding to isolated curli rather than using whole cells, which would not tolerate extreme pH conditions. Solutions of 0.1 mg/mL curli at pH values of 4 to 9.5 (in 80% ethanol) were incubated with 10 μg/mL CR followed by centrifugation to pellet curli and bound CR. Free CR in the supernatant was monitored by UV-Vis spectrophotometry. More CR bound to curli at lower pH values, and CR binding decreased, with more free CR present in the supernatant, as pH values increased from 6 to 9.5 (Fig 2B). Selection of ethanol in the assay was based upon its use in histological screens and assays [30,31]. The general morphology of curli fibers in 80% (v/v) ethanol was not affected as determined by TEM (Fig 2C).

The pH dependence of CR binding to curli is similar to the proton dissociation curve for histidine, and CR binding to β-amyloid fibers formed *in vitro* has been shown to be dependent on the presence of histidine in the protein sequence [32]. Thus, we tested whether histidine might underlie Congo red binding in curli using curli isolated from cells producing: (i) wild type CsgA (as above); (ii) CsgA lacking histidine residues only within the proposed amyloid core (H73G/Δ149), curliΔHis_core_; and (iii) CsgA lacking histidine residues in both the proposed amyloid core and the N-terminus (H32G/H73G/Δ149), curliΔHis_total_. CsgA contains an N-terminal sec signal sequence that is removed upon secretion into the periplasm. Following this, the next N-terminal 22 amino acids are protease sensitive and not thought to be part of the CsgA amyloid core, and includes His32. The proposed amyloid core consists of residues 43–151[33]. As demonstrated in Fig 2B, CR binding to curli fibers isolated from the histidine mutants was comparable to the binding to wild-type fibers. Electron micrographs confirmed that the mutant CsgA proteins assembled into amyloid fibers, although they were somewhat shorter in length from curli isolated from MC4100 (Fig 2C). We note that in the curli preparations, the yield of curli isolated from the His mutants was considerably lower than that of wild type curli isolated from MC4100. Thus, in the His mutants, a preparation using cell lysates was employed to access fibers that still remained cell-associated, whereas a more gentle application of shear forces is sufficient to remove wild-type curli from MC4100.

In summary, CR has been used extensively in microbiological studies to score the production of microbial amyloids as well as polysaccharides and other extracellular factors. However, there is little detail available regarding how CR binds to microbial amyloid fibers. Furthermore, CR inhibits the fibrillization of some amyloidogenic proteins, such as β-amyloid [25], in *in vitro* amyloid polymerization assays. Yet, in microbial systems such as *E*. *coli* and *Salmonella*, CR has not appeared to inhibit amyloid biogenesis *in vivo*. Here, we have demonstrated quantitatively that CR does not inhibit the production of curli in *E*. *coli* when supplemented in nutrient agar at concentrations up to 200 μg/mL, and does not influence the kinetics of curli production.

A tractable feature of microbial amyloids is the presentation of amyloid fibers at the cell surface. Such extracellular display permits facile identification and visualization by electron microscopy, simple whole-cell amyloid-staining using CR or other dyes, and relatively straightforward isolation and purification. Thus, fibers and fiber-dye or fiber-protein complexes can be readily examined using fibers formed natively in their cellular context. Using SPR, we demonstrated that CR binds to immobilized curli with an approximate K_d_ of 2.8 μM. We also discovered that CR binding to curli is pH dependent and is not histidine dependent. This is consistent with a previous study that demonstrated that a synthetic short peptide related to a fragment of CsgA still polymerized and would bind CR despite not containing histidine [34]. Other curli residues may play a role in CR binding to curli such as the aromatic amino acids phenylalanine and tryptophan that may interact with the phenolic rings in CR [35]. In contrast, lysine was shown to be required for CR binding to the fungal prion Het-S [36]. Our comparative analysis on the influence of CR with curliated *E*. *coli* and isolated curli formed natively *in vivo* provides a benchmark from which to compare other curli-binding molecules, mutant curli fibers, curli-type fibers formed through *in vitro* polymerizations, and other functional bacterial amyloids formed *in vivo*.

## Methods

### Curli-associated E. coli sample preparation

Bacteria were grown on YESCA (0.5g/L yeast extract; 10g/L casamino acids) agar plates in the presence or absence of CR at 26°C. A CR (MP Biomedicals, LLC, Cat# 150711, Lot# MR30568) stock solution was prepared as 2 mg/mL in water and sterile-filtered. Designated volumes of CR were added to stirring hot agar medium to achieve the desired final concentration before pouring standard plates (approximately 25mL media per 100 mm petri plate). For the photographed plate in Fig 1A, MC4100 were grown for 48 hours at 26°C on YESCA agar containing 25 μg/mL CR.

### Western blot analysis

The cell-associated curli proteins, CsgA and CsgG, were examined by immunoblot assays as described [37]. Whole-cell samples with equivalent cell number were prepared as cell pellets of 1mL cell culture with an OD_600_ of 1.0. Each pellet was treated with 100 μL hexafluoroisopropanol (HFIP) or formic acid (FA), as indicated, to dissociate curli subunits. HFIP or FA was removed by vacuum centrifugation, and samples were resuspended in SDS-PAGE loading buffer. Protein gel electrophoresis was carried out using 12% SDS-PAGE gels (Invitrogen). Proteins were transferred to 0.2 μm nitrocellulose transfer membranes (Whatman). The polyclonal rabbit antiserum to CsgA or CsgG was used as the primary antibody and horseradish peroxidase (HRP) conjugated goat anti-rabbit antibody (Pierce) was used as the secondary antibody [38].

### Whole-cell Congo red depletion assay

Whole-cell samples were suspended in ice-cold PBS as a dilution series with OD_600_ of 0.2 to 1.0, and CR was added to a final concentration of 10 μg/mL from a filtered stock solution of 1 mg/mL. The cells were incubated with CR for 10 minutes at room temperature with rocking. The cells were pelleted (10,000 *g* for 5 minutes), and the absorbance at 500 nm of the supernatant was measured. For all absorbance experiments, a cell path length of 1 cm was used (Perkin Elmer Lambda 35 UV/Vis Spectrometer). Each sample was prepared and assayed in triplicate. Error bars correspond to the standard deviation of measurements for three separate samples.

### Surface Plasmon Resonance (SPR)-based Biosensor Analysis

The interaction of CR with curli fibers was analyzed by Surface Plasmon Resonance using a BIACORE 3000 biosensor system (GE Healthcare). Analyte (CR) dilutions were performed in HBS-EP (10 mM HEPES, pH 7.4, 150 mM NaCl, 3mM EDTA, and 0.005% v/v Surfactant P20) running buffer. Curli were immobilized onto a CM5 biosensor chip by amine coupling chemistry using N-hydroxysuccinimide (NHS) and N’-(3-dimethylaminopropyl) carbodiimide hydrochloride (EDC). To investigate binding of CR, the diluted analyte was injected over the curli fiber surface. The experiments were performed at 25°C using a flow rate of 50 L/min. For each experiment, at least 5 different concentrations of CR were injected over each experimental and control flow cell for 150 s. Dissociation was allowed to occur at the same flow rate for 180 s in Running Buffer alone. The surface was regenerated after each injection of analyte using two 18 s pulses of 50% DMSO/ 10mM NaOH which led to complete dissociation of the residually bound CR. All data were corrected for non-specific binding by subtracting the signal measured in a control cell lacking immobilized ligand. Both data processing and kinetic fitting were performed using Scrubber software, version 2 (BioLogic Software, Pty., Australia) or BIAevaluation software 4.1 (Biacore).

### Curli production and isolation

Wild-type curli were isolated from YESCA-agar grown MC4100 as previously described [7]. To obtain CsgA lacking histidines or lacking only core histidines, appropriate plasmids (pMC3-H32G-H73G-Δ149 and pMC3-H73G-Δ149, respectively) were generated using pMC3 (Chapman Laboratory) as a template (GenScript, New Jersey, USA). Protein expression was performed in *E*.*coli* strain LSR12 grown in Luria broth at 37°C. Protein expression was induced with 0.25 mM ITPG for 60 minutes when cell densities reached an OD_600_ of ~1. Cells were harvested by centrifugation (10,000 *g*, 20 minutes, 4°C). The cell pellet was frozen and then resuspended in lysis buffer (8 M GdnHCl). Cells were lysed by sonication (6x 10 s bursts with 15 s cooling period on ice between each burst) and centrifuged (10,000 *g*, 20 minutes, 4°C) to remove cellular debris. The supernatant was dialyzed overnight against 50 mM Na_2_HPO_4_ (3.5 MWCO), and then centrifuged (13,000 *g*, 30 minutes, 20°C). The pellet was resuspended in 10 mM Tris, pH 7.4 with 4% (w/v) final concentration SDS and incubated overnight, rocking at room temperature. The CsgA-H73G-Δ149 fibrils were initially pelleted (13,000 g, 60 minutes, 20°C) and then resuspended in 10 mM Tris (pH 7.4). The curli were pelleted again (30,000 *g*, 30 minutes, 20°C) and resuspended in 10 mM Tris (pH 7.4); this final washing step was repeated approximately four times until the SDS was removed. The final curli pellet was resuspended in 5 mL of water. The concentration of the curli solution was determined using the BCA Protein Assay (Thermo Scientific Pierce BCA Protein Assay Kit). CR was quantified by measuring the absorbance at 500 nm in 10 mM Tris-HCl at pH 7.5, with an extinction coefficient of 5 x 10^4^ M^-1^ cm^-1^.

### Transmission electron microscopy (TEM)

Negative staining TEM was performed on curli samples. Samples were applied to 300-mesh copper grids coated with Formvar film (Electron Microscopy Sciences, Hatfield, PA) for 2 min, rinsed in deionized water, negatively stained with 2% uranyl acetate for 90 s, and air-dried. Microscopy was performed on the JEM-1400 (JEOL, LLC).

### Curli-only Congo red depletion assay

Curli (0.1 mg/mL) were suspended in solutions of nominal pH values of 4 to 9.5, in 80% (v/v) ethanol and 5 mM buffer (glycine at pH 4.0 and 5.0; 4-morpholineethanesulfonic acid (MES) at pH 6.0 and 6.5; and Tris(hydroxymethyl)-aminomethane (TRIS) at pH 7.0 and 9.5). For the pH-dependence assays, curli (0.1 mg/mL) were incubated with 10 μg/mL CR (from 1 mg/mL aqueous stock solution) for 10 minutes, rocking at room temperature before centrifugation (13,000 *g* for 10 minutes) to remove curli and bound CR. The supernatant absorbance was measured at 500 nm. Each sample was prepared and assayed in triplicate with the exception of CsgA-H32G-H73G-Δ149 fibrils, which was only a single sample due to limited sample size. Error bars correspond to the standard deviation of measurements for three separate samples.

## Supporting Information

S1 Fig Congo red and *E*. *coli*. Supplementation of the agar with CR at concentrations of 25μg/mL does not affect curli production on a per cell basis as assessed by Western blot analysis for any of the *E*. *coli* clinical isolates that were tested. The blots were probed for CsgA, which is the main curli fiber subunit. Additionally, in all cases, formic acid was required to depolymerize curli fibers into its SDS-soluble subunits.
(TIF)

S2 Fig Testing regeneration conditions for SPR. Suitable regeneration conditions (RC) were required to remove CR from curli fibers during the SPR measurements. The conditions tested are shown in the figure. CR was incubated with either (A) whole-cells or (B) isolated curli, and centrifuged to obtain a pellet. The pellet was then resuspended in the RC before centrifugation. The pellets before and after incubation in the RC are shown. For the successful conditions, CR was removed from the pelleted whole-cells or isolated curli. A few successful conditions were identified: 100% DMSO; 50% DMSO/10 mM NaOH; and 100% DMF. We selected 50%DMSO/10mM NaOH as the RC for our SPR experiments as it was most compatible with the SPR instrument and protocols.
(JPG)

## References

[1] Olsen A, Jonsson A, Normark S. Fibronectin binding mediated by a novel class of surface organelles on Escherichia coli. Nature. 1989;338(6217):652–5. 2649795 10.1038/338652a0

[2] Collinson SK, Emody L, Muller KH, Trust TJ, Kay WW. Purification and characterization of thin, aggregative fimbriae from Salmonella enteritidis. J Bacteriol. 1991;173(15):4773–81. 1677357 10.1128/jb.173.15.4773-4781.1991 PMC208156

[3] Chapman MR, Robinson LS, Pinkner JS, Roth R, Heuser J, Hammar M, et al Role of Escherichia coli curli operons in directing amyloid fiber formation. Science. 2002;295(5556):851–5. 11823641 10.1126/science.1067484 PMC2838482

[4] Barnhart MM, Chapman MR. Curli biogenesis and function. Annu Rev Microbiol. 2006;60:131–47. 16704339 10.1146/annurev.micro.60.080805.142106 PMC2838481

[5] Capstick DS, Jomaa A, Hanke C, Ortega J, Elliot MA. Dual amyloid domains promote differential functioning of the chaplin proteins during Streptomyces aerial morphogenesis. Proc Natl Acad Sci USA. 2011;108(24):9821–6. 10.1073/pnas.1018715108 21628577 PMC3116418

[6] Romero D, Aguilar C, Losick R, Kolter R. Amyloid fibers provide structural integrity to Bacillus subtilis biofilms. Proc Natl Acad Sci USA. 2010;107(5):2230–4. 10.1073/pnas.0910560107 20080671 PMC2836674

[7] Schwartz K, Syed AK, Stephenson RE, Rickard AH, Boles BR. Functional Amyloids Composed of Phenol Soluble Modulins Stabilize Staphylococcus aureus Biofilms. Plos Pathog. 2012;8(6). 10.1371/journal.ppat.1002744 PMC3369951 22685403

[8] Alteri CJ, Xicohtencatl-Cortes J, Hess S, Caballero-Olin G, Giron JA, Friedman RL. Mycobacterium tuberculosis produces pili during human infection. Proc Natl Acad Sci USA. 2007;104(12):5145–50. 17360408 10.1073/pnas.0602304104 PMC1817835

[9] Dueholm MS, Petersen SV, Sonderkaer M, Larsen P, Christiansen G, Hein KL, et al Functional amyloid in Pseudomonas. Mol Microbiol. 2010 77, 1009–1020. 10.1111/j.1365-2958.2010.07269.x 20572935

[10] Wu C, Lim JY, Fuller GG, Cegelski L. Quantitative analysis of amyloid-integrated biofilms formed by uropathogenic Esherichia coli at the air-liquid interface. Biophys J. 2012;103(3):464–71. 10.1016/j.bpj.2012.06.049 22947862 PMC3414876

[11] Lembré P, Di Martino P, Vendrely C. Amyloid peptides derived from CsgA and FapC modify the viscoelastic properties of biofilm model matrices. Biofouling. 2014;30(4):415–426. 10.1080/08927014.2014.880112 24592895

[12] Goyal P, Krasteva PV, Van Genven N, Gubellini F, Van den Broeck I, Troupiotis-Tsailaki A, et al Structural and mechanistic insights into the bacterial amyloid secretion channel CsgG. Nature. 2014;516(7530):250–3. 10.1038/nature13768 25219853 PMC4268158

[13] Robinson LS, Ashman EM, Hultgren SJ, Chapman MR. Secretion of curli fibre subunits is mediated by the outer membrane-localized CsgG protein. Mol Microbiol. 2006;59(3):870–81. 16420357 10.1111/j.1365-2958.2005.04997.x PMC2838483

[14] Nenninger AA, Robinson LS, Hultgren SJ. Localized and efficient curli nucleation requires the chaperone-like amyloid assembly protein CsgF. Proc Natl Acad Sci USA. 2009;106(3):900–5. 10.1073/pnas.0812143106 19131513 PMC2630086

[15] Chiti F, Dobson CM. Protein misfolding, functional amyloid, and human disease. Annu Rev Biochem. 2006;75:333–66. 16756495 10.1146/annurev.biochem.75.101304.123901

[16] Eanes ED, Glenner GG. X-Ray Diffraction Studies on Amyloid Filaments. J Histochem Cytochem. 1968;16(11):673–7. 5723775 10.1177/16.11.673

[17] Bonar L, Cohen AS, Skinner MM. Characterization of Amyloid Fibril as a Cross-Beta Protein. P Soc Exp Biol Med. 1969;131(4):1373–5. 10.3181/00379727-131-34110 5812002

[18] Manning M, Colon W. Structural basis of protein kinetic stability: Resistance to sodium dodecyl sulfate suggests a central role for rigidity and a bias toward beta-sheet structure. Biochemistry-Us. 2004;43(35):11248–54. 10.1021/bi0491898 15366934

[19] Sipe JD. Amyloidosis. Annu Rev Biochem. 1992;61:947–75. 1497327 10.1146/annurev.bi.61.070192.004503

[20] Westermark GT, Johnson KH, Westermark P. Staining methods for identification of amyloid in tissue. Amyloid, Prions, and Other Protein Aggregates. 1999;309:3–25. 10.1016/s0076-6879(99)09003-5 10507013

[21] Reinke AA, Gestwicki JE. Insight into amyloid structure using chemical probes. Chem Biol Drug Des.77(6):399–411. 10.1111/j.1747-0285.2011.01110.x 21457473 PMC3097318

[22] McCrate OA, Zhou X, Cegelski L. Curcumin as an Amyloid-indicator Dye in E. coli. Chemical Communications. 2013; 49: 4193–4195. 10.1039/c2cc37792f 23287899 PMC3633639

[23] Garofalo CK, Hooton TM, Martin SM, Stamm WE, Palermo JJ, Gordon JI, et al Escherichia coli from urine of female patients with urinary tract infections is competent for intracellular bacterial community formation, Infect. Immun. 2007;75: 52–60. 17074856 10.1128/IAI.01123-06 PMC1828379

[24] Lim JY, Pinkner JS, Cegelski L. Community Behavior and Amyloid-associated Phenotypes among a Panel of Uropathogenic E. coli. Biochem. Biophys. Res. Commun. 2014; 443: 345–350. 10.1016/j.bbrc.2013.11.026 24239885 PMC3932320

[25] Podlisny MB, Walsh DM, Amarante P, Ostaszewski BL, Stimson ER, Maggio JE, et al Oligomerization of Endogeneous and Synthetic Amyloid Beta-Protein at Nanomolar Levels in Cell Culture and Stabilization of Monomer by Congo Red. Biochemistry-Us. 1998;37:3602–11. 10.1021/bi972029u 9521679

[26] Gophna U, Barlev M, Seijffers R, Oelschlager TA, Hacker J, Ron EZ. Curli fibers mediate internalization of Escherichia coli by eukaryotic cells. Infect Immun. 2001;69(4):2659–65. 11254632 10.1128/IAI.69.4.2659-2665.2001 PMC98204

[27] Cherny I, Rockah L, Levy-Nissenbaum O, Gophna U, Ron EZ, Gazit E. The formation of Escherichia coli curli amyloid fibrils is mediated by prion-like peptide repeats. J Mol Biol. 2005;352(2):245–52. 16083908 10.1016/j.jmb.2005.07.028

[28] Klunk WE, Jacob RF, Mason RP. Quantifying amyloid by Congo red spectral shift assay. Method Enzymol. 1999;309:285–305. 10.1016/s0076-6879(99)09021-7 10507031

[29] Kai-Larsen Y, Luthje P, Chromek M, Peters V, Wang X, Holm A, et al Uropathogenic Escherichia coli modulates immune responses and its curli fimbriae interact with the antimicrobial peptide LL-37. Plos Pathog.6(7):e1001010 10.1371/journal.ppat.1001010 20661475 PMC2908543

[30] Inouye H, Nguyen J, Fraser P, Shinchuk L, Packard A, Kirschner D. Histidine residues underlie Congo red binding to A beta analogs. Amyloid. 2000;7(3):179–88. 11019858 10.3109/13506120009146832

[31] Eisert R, Felau L, Brown LR. Methods for enhancing the accuracy and reproducibility of Congo red and thioflavin T assays. Analytical biochemistry. 2006;353(1):144–6. 16620754 10.1016/j.ab.2006.03.015

[32] Inouye H, Nguyen JT, Fraser PE, Shinchuk LM, Packard AB, Kirschner DA. Histidine residues underlie Congo red binding to A beta analogs. Amyloid-International Journal of Experimental and Clinical Investigation. 2000;7(3):179–88. 10.3109/13506120009146832 11019858

[33] Wang X, Smith DR, Jones JW, Chapman MR. In vitro polymerization of a functional Escherichia coli amyloid protein. The Journal of biological chemistry. 2007;282(6):3713–9. 17164238 10.1074/jbc.M609228200 PMC2838475

[34] Lembré P, Vendrely C, Di Martino P. Amyloid Fiber Formation by Synthetic Peptides Derived from the Sequence of the Protein CsgA of Escherichia coli. Prot Pept Lett. 2013;20:942–946. 10.2174/0929866511320080012 23360366

[35] Porat Y, Abramowitz A, Gazit E. Inhibition of amyloid fibril formation by polyphenols: structural similarity and aromatic interactions as a common inhibition mechanism. Chem Biol Drug Des. 2006;67(1):27–37. 16492146 10.1111/j.1747-0285.2005.00318.x

[36] Schutz AK, Soragni A, Hornemann S, Aguzzi A, Ernst M, Bockmann A, et al The amyloid-Congo red interface at atomic resolution. Angew Chem Int Ed Engl. 2011;50(26):5956–60. 10.1002/anie.201008276 21591034

[37] Cegelski L, Pinkner JS, Hammer ND, Cusumano CK, Hung CS, Chorell E, et al Small-molecule inhibitors target Escherichia coli amyloid biogenesis and biofilm formation. Nat Chem Biol. 2009;5(12):913–9. 10.1038/nchembio.242 19915538 PMC2838449

[38] Nenninger AA, Robinson LS, Hammer ND, Epstein EA, Badtke MP, Hultgren SJ, et al CsgE is a curli secretion specificity factor that prevents amyloid fibre aggregation. Molecular Microbiology. 2011;81(2):486–99. 10.1111/j.1365-2958.2011.07706.x 21645131 PMC3134098

