# Supplementary figures and images for: Congo Red Interactions with Curli-Producing E. coli and Native Curli Amyloid Fibers
Source: PLoS One. 2015 Oct 20;10(10):e0140388. doi: 10.1371/journal.pone.0140388 (PMC4618944; doi:10.1371/journal.pone.0140388)

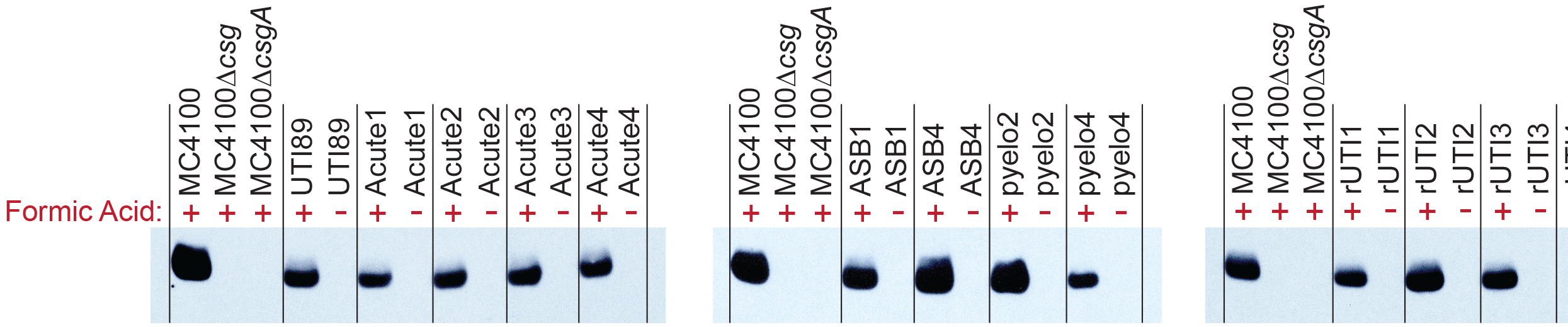

Supplement: S1 Fig — Supplementation of the agar with CR at concentrations of 25μg/mL does not affect curli production on a per cell basis as assessed by Western blot analysis for any of the E. coli clinical isolates that were tested. The blots were probed for CsgA, which is the main curli fiber subunit. Additionally, in all cases, formic acid was required to depolymerize curli fibers into its SDS-soluble subunits. (TIF) [file pone.0140388.s001.tif]

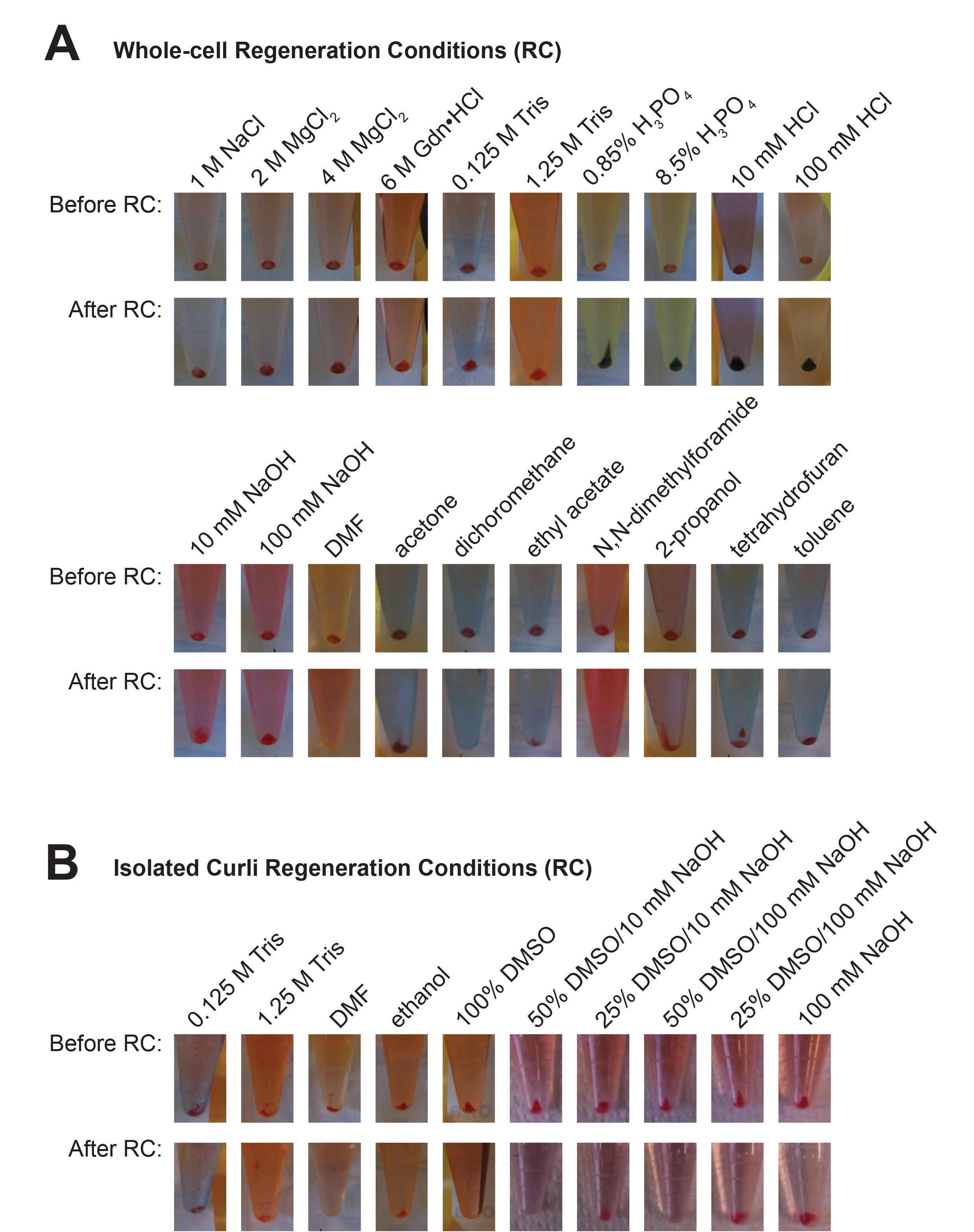

Supplement: S2 Fig — Suitable regeneration conditions (RC) were required to remove CR from curli fibers during the SPR measurements. The conditions tested are shown in the figure. CR was incubated with either (A) whole-cells or (B) isolated curli, and centrifuged to obtain a pellet. The pellet was then resuspended in the RC before centrifugation. The pellets before and after incubation in the RC are shown. For the successful conditions, CR was removed from the pelleted whole-cells or isolated curli. A few successful conditions were identified: 100% DMSO; 50% DMSO/10 mM NaOH; and 100% DMF. We selected 50%DMSO/10mM NaOH as the RC for our SPR experiments as it was most compatible with the SPR instrument and protocols. (JPG) [file pone.0140388.s002.jpg]
